# *In silico* analysis and verification of S100 gene expression in gastric cancer

**DOI:** 10.1186/1471-2407-8-261

**Published:** 2008-09-16

**Authors:** Ji Liu, Xue Li, Guang-Long Dong, Hong-Wei Zhang, Dong-Li Chen, Jian-Jun Du, Jian-Yong Zheng, Ji-Peng Li, Wei-Zhong Wang

**Affiliations:** 1 Department of Gastrointestinal Surgery, Xijing Hospital, The Fourth Military Medical University, Xi'an, 710032, PR China; 2 Department of Ultrasonic Diagnosis, Xijing Hospital, The Fourth Military Medical University. Xi'an, 710032, PR China

## Abstract

**Background:**

The S100 protein family comprises 22 members whose protein sequences encompass at least one EF-hand Ca^2+ ^binding motif. They were involved in the regulation of a number of cellular processes such as cell cycle progression and differentiation. However, the expression status of S100 family members in gastric cancer was not known yet.

**Methods:**

Combined with analysis of series analysis of gene expression, virtual Northern blot and microarray data, the expression levels of S100 family members in normal and malignant stomach tissues were systematically investigated. The expression of S100A3 was further evaluated by quantitative RT-PCR.

**Results:**

At least 5 S100 genes were found to be upregulated in gastric cance by in silico analysis. Among them, four genes, including S100A2, S100A4, S100A7 and S100A10, were reported to overexpressed in gastric cancer previously. The expression of S100A3 in eighty patients of gastric cancer was further examined. The results showed that the mean expression levels of S100A3 in gastric cancer tissues were 2.5 times as high as in adjacent non-tumorous tissues. S100A3 expression was correlated with tumor differentiation and TNM (Tumor-Node-Metastasis) stage of gastric cancer, which was relatively highly expressed in poorly differentiated and advanced gastric cancer tissues (*P *< 0.05).

**Conclusion:**

To our knowledge this is the first report of systematic evaluation of S100 gene expressions in gastric cancers by multiple in silico analysis. The results indicated that overexpression of S100 gene family members were characteristics of gastric cancers and S100A3 might play important roles in differentiation and progression of gastric cancer.

## Background

Gastric cancer is the second most common cause of cancer death worldwide. Environmental and genetic factors are both important in gastric carcinogenesis [1,2]. In the past two decades, much progress has been made in identifying genes involved in the development of gastric cancer. These identified genes are useful in understanding the pathogenesis of gastric cancer and defining its molecular signature. They can also serve as biomarkers for early diagnosis and targets for drug development.

Recently, large-scale gene expression analyses have emerged as important tools for screening genes related to cancer [3]. The two experimental technologies available for large-scale gene expression analysis are: 1) DNA sequencing-based serial analysis of gene expression (SAGE) and expressed sequence tag (EST) approaches and 2) dot-blot based microarray analysis. Multiple bioinformatics infrastructures have been established to compile data derived from these techniques. Among them, the Cancer Genome Anatomy Project (CGAP) and Gene Expression Omnibus (GEO) are two important networks[4,5]. Previous applications of data mining using CGAP and GEO resource have led to the identification of several novel or known cancer-related genes [6,7].

The S100 protein family comprises 22 members whose protein sequences encompass at least one EF-hand Ca2+ binding motif[8]. S100 proteins are localized in the cytoplasm and/or nucleus of a wide range of cells, and involved in the regulation of a number of cellular processes such as cell cycle progression and differentiation. Seventeen S100 family members are located as a cluster on chromosome 1q21–22, a region frequently rearranged in several tumors. In addition, molecular analysis has revealed that several S100s, including S100A2, S100A4, S100A7 and S100A10, exhibit altered expression levels in gastric cancer[9,10]. So it is interesting to systematically investigate the expression of other members of S100 family in normal and gastric cancer tissues.

In this study, we utilized the databases and analytical approaches available from the Gene Expression Omnibus and Cancer Genome Anatomy Project to systematically analyze the expression of 22 S100 genes in normal and cancerous stomach tissues. The independent SAGE and microarray datasets were screened for S100 gene expression patterns. We provided evidence that at least five S100 genes were upregulated in gastric cancer and further experimentally verified the upregulation of S100A3 by quantitative RT-PCR.

## Methods

### SAGE analysis

SAGE measures the number of tags that represent the transcriptional products of a gene. The data produced by SAGE technology is a list of tags with their corresponding count values. All publicly available SAGE data collected in GEO website up to January 2008 were used for analysis of S100 gene expression. Both NlaIII and Sau3A tags from SAGEmap  were mapped to UniGene clusters . The reliable UniGene clusters matched to S100 tags were adopted. These sequence tags were then used to determine the levels of expression of 22 S100 genes in 2 normal and 8 gastric cancer libraries. A list of the tags used for analysis was provided in Table 1 and detailed information about these libraries are available from the GEO website. Analyses were performed by comparing the average number of S100 tags in libraries of normal mucosa with that in libraries of gastric cancer. Difference of >3-fold change will be considered as positive.

**Table 1:** SAGE analysis of S100 genes expression in normal and gastric cancer libraries

Gene name	Unigene cluster	SAGE tag	Normal (tpm*)	Cancer (tpm*)
S100A2	516484	GATCTCTTGG	0.0	98.1
S100A3	433168	TCTCCCACAC	0.0	2.8
S100A4	81256	ATGTGTAACG	0.0	207.8
S100A6	275243	CCCCCTGGAT	245.9	865.9
S100A7	112408	GAGCAGCGCC	0.0	143.8
S100A8	416073	TACCTGCAGA	0.0	549.2
S100A9	112405	GTGGCCACGG	0.0	637.0
S100A10	143873	AGCAGATCAG	263.6	1890.5
S100A12	19413	GATTTTTAAA	0.0	13.9
S100A16	515714	AGCAGGAGCA	0.0	130.6

Only S100s that show positive matches are shown in this table. *tags per million;

### Virtual Northern

In the CGAP database, virtual Northern blot analysis enables researchers to view the expression of a specific gene in all EST and SAGE libraries[4]. Through the Gene Finder tool , some organized information about a particular gene can be found by querying either the unique gene identifier or a key word. Included in the available information are the EST and SAGE vNorthern expressional patterns across all available libraries classified according to their tissue origins. The SAGE data collected in CGAP include 5 tissue libraries and 2 xenograft libraries of gastric cancer, as well as 3 normal libraries of stomach, which were partially different from that collected in GEO mentioned above. S100 genes whose expression in EST and SAGE libraries of gastric cancer was both >3 fold as much as those in normal stomach were designated as positive ones.

### Microarray analysis

At present, five microarray datasets (Table 2) containing data from normal and gastric cancer tissues were available in GEO website . The dataset GSE2669, GSE2701 and GSE3438 contributed by Boussioutas A[11], Chen X[12] and Kim S[13] respectively were chosen for conducting microarray analysis because these three datasets contained relatively more cases of normal and gastric cancer (10 normal ones and 64 cancers for Boussioutas A et al; 22 normal ones and 90 cancers for Chen X; 50 normal ones and 50 cancers for Kim S). More details about the samples and microarray analysis can be found from the GEO website. Difference was considered significant when *p *< 0.05. S100 genes whose expression was both highly in three groups of gastric cancer tissues were designated as positive ones.

**Table 2:** Microarray datasets of gastric cancer collected in Gene Expression Omnibus website.

	*Cases*		*Array*	*Spot*	*Supplier*
				
	Normal	Cancer			
GSE2637	3	55	cDNA	13 k/17 K	Aggarwal A et al
GSE2685	8	22	Oligo- nucleotide	~7.2 K	Hippo Y et al
GSE2669	10	64	cDNA	~7.4 K	Boussioutas A et al
GSE2701	22	90	cDNA	44 K	Chen X et al
GSE3438	50	50	cDNA	14 K	Kim S et al

### Tissue Collection

Eighty patients with gastric cancer who underwent surgery in our hospital from September of 2004 to March of 2007 were enrolled in this study. The resected tumor and adjacent non-tumorous tissue specimens were immediately frozen in liquid nitrogen and kept at 70°C until RNA extraction. The diagnosis of both gastric cancer and normal gastric mucosa was clinically and pathologically proved. The patient's sex, age, tumor size, TNM stage, depth of wall invasion, microscopic subtype, and status of lymph node metastasis were obtained from surgical and pathological records. The protocols used in this study were approved by the Hospital's Protection of Human Subjects Committee. Patients providing fresh surgical tissue for the study signed informed consent.

### Quantitative RT-PCR

Total RNA (mRNA) was extracted from gastric cancer and adjacent non-tumorous tissues according to the manufacturer's recommendations of TRIzol reagent (Invitrogen, Carlsbad, CA). 1 μg sample of total RNA was reverse transcribed to complementary DNA (cDNA) with oligo (dT) primers. The sense and antisense primers for S100A3 were designed according to the mRNA sequence (GenBank accession number NM_002960.1). We used amplified PCR fragments spanning different exons to prevent amplification of contaminated genomic DNA. The sense primer was 5'-GACCATCTGGTTCAGGTTCC-3' and the antisense primer was 5'-ACATTCCCGAAACTCAGTCG-3'. The PCR products were 200 bp in size. The housekeeping gene GAPDH was used as an internal control. The sense primer was 5'-CCAGGTGGTCTCCTCTGACTT-3' and the antisense primer was 5'-GTTGCTGTAGCCAAATTCGTTGT-3'. The PCR products were 130 bp in size.

The standard curve was produced by measuring the crossing-point of each standard value (6-fold serially diluted cDNAs of cardiac muscle, in which the content of S100A3 was relatively abundant) and plotting them against the logarithmic value of the concentrations. Standard curve samples were included in each run. Quantitative real-time RT-PCR was performed using an ABI PRISM 7000 sequence detection system (Applied Biosystems, Foster City, CA). The RT-PCR was carried out in a total volume of 30 μL. The reaction mixture included 1× buffer, 200 μmol/L of deoxy-ribonucleoside triphosphates (dNTPs) (Invitrogen), 0.3 μmol/L of sense and antisense primers, 1 U of Takara ExTaq Hotstart Taq (TaKaRa Biotechnology), 0.6 μL of 5-carboxy-x-rhodamine (ROX) reference dye, and 2 μL of cDNA. The PCR cycle involved 2 minutes at 95°C followed by 40 amplification cycles of denaturation at 94°C for 30 seconds, annealing at 58°C (for detection of GAPDH) or 55°C (for detection of S100A3) for 30 seconds, and elongation at 72°C for 1 minute. The relative quantitation of both S100A3 and GAPDH was determined by the comparative CT (thermal cycle) method. The values of S100A3 mRNA expression were normalized according to the expression of GAPDH. Each assay was repeated three times to verify the results, and the ratio of mRNA expression value of gastric cancer tissues to adjacent non-tumorous tissues was used for subsequent analysis.

### Statistical analysis

For continuous variables, the data were expressed as the means+/-SD. Expression data of S100 genes in microarray datasets was retrieved and the differences in expression levels between normal and gastric cancer tissues were determined by student T test. The association between relative expression ratios of S100A3 and clinical features was analyzed by Mann-Whitney test. All data were analyzed using the SPSS11.0 software package (SPSS, Chicago, USA) and the difference was considered significant when p < 0.05.

## Results

### 1. SAGE and virtual Northern blot analysis of S100 genes expression in gastric cancer

There are 2 SAGE libraries of normal gastric mucosa and 8 SAGE libraries of gastric cancer tissues available in GEO website (GSE545 and GSE14). These libraries were provided by two different labs [14,15]. The reliable tags of 20 S100 genes were extracted from SAGEmap website and used to search the SAGE data. 10 genes were found to be highly expressed in gastric cancer tissues according to the setting criteria of >3-fold difference (Table 1). In these 10 genes, only S100A6 and S100A10 could be detectable in normal gastric mucosa tissues, which also had top average density in gastric cancer (865.9 tpm and 1890.5 tpm respectively). The other 8 genes, including S100A2, S100A3, S100A4, S100A7, S100A8, S100A9, S100A12 and S100A16, had different average density ranging from 2.8 tpm to 637.0 tpm, none of which were expressed in normal gastric mucosa tissues. No significant difference in expression between normal and cancerous tissues could be found on S100A11, S100A14 and S100P (data not shown). We then used virtual Northern blot to analyze the expression of S100 genes (Table 3). Six genes were confirmed to be upregulated in gastric cancer tissues. The positive S100A6, S100A8, and S100A16 identified by SAGE analysis were shown to have no significant difference in expression between normal and gastric cancer libraries when conducting EST virtual Northern Blot. S100A13, not detectable in SAGE libraries, was shown to be highly expressed in gastric cancer tissues by EST virtual Northern blot. S100A3 and S100A12 could not be detected by virtual Northern blot.

**Table 3:** Virtual Northern blot analysis of S100 genes expression in normal and gastric cancer libraries

	EST tags (tpm*)		SAGE tags (tpm*)	
	
	Normal	Cancer	Normal	Cancer
S100A2	0.0	25.9	0.0	200.5
S100A3	0.0	0.0	0.0	0.0
S100A4	52.3	293.7	0.0	168.4
S100A7	0.0	0.0	0.0	304.8
S100A9	0.0	8.6	0.0	1339.4
S100A10	0.0	181.4	272.3	1483.7
S100A12	0	0	0	0
S100A13	0.0	43.2	0.0	0.0

Only S100s that show positive matches are shown in this table. *tags per million;

### 2. Microarray analysis of S100 genes expression in gastric cancer

Microarray analysis was conducted to further verify the overexpression of S100 genes in gastric cancer. 8, 11 and 7 S100 genes could be found in GSE2669, GSE2701 and GSE3438 datasets respectively (Table 4). Both S100A2 and S100A10 genes were shown to be upregulated in gastric cancer of all three datasets. S100A3 was demonstrated to be overexpressed in gastric cancer by dataset GSE2669 and GSE2701, which did not existed in dataset GSE3438. S100A4, S100A6 and S100A7 were shown to be upregulated in gastric cancer in only one dataset but did not exist in the other two datasets. However, expression of S100A8 and S100A9 had no significant difference between normal and cancerous tissues in dataset GSE2701. The differential expression tendency of S100A12 in GSE2701 was contrary to that of SAGE analysis (Table 1).

**Table 4:** Microarray analysis of S100 genes expression in normal and gastric cancer tissues

	GSE3438			GSE2669			GSE2701		
	Normal*	Cancer*	*p*	Normal*	Cancer*	*p*	Normal*	Cancer*	*P*

S100A2	-0.22	0.00	0.00	1.03	1.35	0.01	-0.95	-0.36	0.00
S100A3	#	#	#	0.84	1.55	0.03	-0.14	0.05	0.03
S100A4	-0.23	0.30	0.00	#	#	#	#	#	#
S100A6	-0.43	0.27	0.00	#	#	#	#	#	#
S100A7	#	#	#	#	#	#	-1.00	-0.35	0.00
S100A8	#	#	#	0.32	0.69	0.01	0.66	0.70	0.81
S100A9	#	#	#	1.25	3.48	0.00	0.42	0.79	0.13
S100A10	-0.50	0.42	0.00	0.46	1.36	0.00	0.19	1.22	0.00
S100A12	#	#	#	#	#	#	0.50	0.21	0.01

Only S100s that show positive matches are shown in the table. *data extracted from microarray datasets; # data not exist in the dataset.

Taken together, 5 genes were demonstrated to be highly expressed in gastric cancer by all three in silico analysis approaches. They were S100A2, S100A3, S100A4, S100A7 and S100A10. Among these 5 genes, S100A3 was the only one which was not yet reported to be related to gastric cancer previously. Next we evaluated the expression of S100A3 in gastric cancer tissues by quantitative RT-PCR.

### 3. Verification of S100A3 overexpression in gastric cancer by quantitative RT-PCR

To investigate whether S100A3 was overexpressed in gastric cancer, we examined the mRNA expression of S100A3 in gastric cancer tissues and corresponding adjacent non-tumorous tissues of 80 patients by quantitative RT-PCR. The relatively mean expression level of S100A3 in gastric cancer was 2.52 ± 1.45 when compared to adjacent non-tumorous tissues (*p *= 0.01). Correlations of S100A3 mRNA expressions with the clinical features were further analyzed. The results showed that S100A3 mRNA expression did not correlated with gender, age, tumor size, depth of wall invasion, microscopic subtypes or lymph node metastasis with a statistic p > 0.05 in each parameter (Table 5). However, we found that the expression of S100A3 mRNA was correlated with tumor differentiation and Tumor-Node-Metastasis stage. The S100A3 expression levels in well- and moderate-differentiated tumor tissues were both significantly lower than that in poorly differentiated ones (*p *< 0.05). S100A3 expression in TNM stage I and II was also lower than that in stage III and IV (*p *= 0.04). All these data demonstrated that S100A3 was overexpressed in gastric cancer specimens and might be related to the differentiation and development of gastric cancer.

**Table 5:** Correlation of S100A3 mRNA expression with clinicalpathological parameters in patients with gastric carcinoma.

**Variable**	mRNA expression					*P*
		
	Number	N/T >4	N/T 2–4	N/T 1–2	N/T <1	
**Gender**						
Male	61	11	34	9	8	0.17
Female	19	3	5	8	2	
**Age (y)**						
<65	54	10	31	6	7	0.08
>65	26	4	8	11	3	
**Tumor differentiation**						
Well^1^	3	0	0	1	2	0.22^a^
Moderate^2^	33	8	7	12	6	0.03^b^
Poor^3^	44	6	32	4	2	0.01^c^
**Tumor size (cm)**						
<5.0	41	6	23	5	7	0.96
>5.0	39	8	16	12	3	
**Depth of wall invasion**						
Mucosa, submucosa^1^	6	2	1	2	1	0.45^a^
Muscularis propria^2^	18	6	8	4	0	0.33^b^
Subserosa, serosa^3^	56	10	34	6	6	0.72^c^
**Stage**						
I + II	19	2	7	5	5	0.04
III + IV	61	12	32	12	5	
**Microscopic subtypes**						
Intestinal^1^	57	10	29	11	7	0.87^a^
Diffuse^2^	18	4	8	4	2	0.26^b^
Atypical^3^	5	0	2	2	1	0.21^c^
**Lymph node metastasis**						
Yes	64	11	35	11	7	0.16
No	16	3	4	6	3	

^a^*P*: 1 *VS *2; ^b^*P*: 2 *VS *3; ^c^*P*: 1 *VS *3.

## Discussion

Although S100 family members have a common structure and are mainly localized in a specific region of chromosome 1, they all have very unique expression patterns in normal or pathological tissues. We systematically investigated the expression of S100 family members in gastric cancer tissues by combining analysis of SAGE, virtual Northern blot and microarray data. It has been reported that cross-hybridization errors may happen in microarray analysis when sequence similarity exceeds 75%. However, the mRNA sequence similarity of S100 family members was 4%–67%, so the possibility of cross-hybridization of S100 genes on all the three chips analyzed in the present study would be relatively small. Furthermore, Both SAGE technology and EST virtual Northern blot were based on DNA sequencing and were thought to be reliable methods in gene expression evaluation. Combining multiple database and analytical approaches mentioned above, the possibility of artifacts would be greatly reduced. In the present study, 5 S100 genes were demonstrated to be upregulated in gastric cancer by combining analysis, among which 4 genes were reported previously, indicating the validity of in silico analysis strategy we used in this work and the possibly important role of S100 genes in development and progression of gastric cancer.

In recent years, many S100 family members have been shown to be differentially regulated in diverse malignancies. Although the action mechanisms of S100s and the functional implications of their altered expression remained to be determined, several studies have demonstrated that overexpression of S100 proteins shows great clinical implications for the diagnosis and staging of human tumors, as well as for the prediction of prognosis.

It has been reported that S100A2 was highly expressed in non-small cell lung cancer, esophageal squamous cell carcinoma, laryngeal squamous cell carcinoma, ovarian serous papillary carcinomas, as well as gastric cancer. S100A2 was also shown to be a predictor of distant metastasis and survival rate in early-stage non-small cell lung cancer[16]. S100A4 was found to be overexpressed in bladder cancer and could be served as a predictor of tumor progression [17]. Many studies have showed that S100A7 had an increased expression in breast cancer. The overexpression of S100A7 was related to higher TNM stages of breast cancer and in estrogen receptor-negative invasive breast cancers, S100A7 expression was associated with poor outcome [18]. S100A7 overexpression was also associated with increased malignancy of breast cancer, which may occur through stimulation of Jab1 activity. In addition, S100A10 was identified as an upregulated gene in squamous non-small cell lung cancers and esophageal squamous cell carcinoma by microarray technology. All these 4 S100 genes were also shown to be upregulaetd in gastric cancers previously [9,10], which was further confirmed in the present work.

S100 genes may be downregulated in some other cancers. For example, S100A6 was lowly expressed in prostate cancers, which might be related to promoter hypermethylation of S100A6. Another example is S100A2. It had a reduced expression in prostate and oral cancer and was regarded as a potential tumor suppressor. S100A2 can interact with C terminus of p53 and then enhance the transcriptional activity of p53. This cell cycle-dependent p53-S100A2 interaction might mediate the inhibiting effect of S100A2 on cancer. These results suggested that S100 genes might play different roles during development of different cancers.

S100A3, a protein correlated with the development of hair follicle, had been proven to be overexpressed in tumors. For example, the levels of expression of the S100A3 proteins differed markedly in the astrocytic tumour tissue in relation to the tumour types and grades[19]. The present work confirmed for the first time that S100A3 was upregulated in gastric cancer and associated with the poor differentiation and higher TNM stage of gastric cancer cells. However, the role of S100A3 in differentiation and progression of gastric cancer needed to be further investigated.

## Conclusion

Our work suggested that in silico analysis is a valid strategy for discovering differentially expressed genes in gastric cancer, and S100A3 was a novel overexpressed gene in gastric cancer cells and might play an important role during the differentiation and progression of gastric cancer.

## Abbreviations

SAGE: Serial Analysis of Gene Expression; EST: Expressed Sequence Tag; CGAP: Cancer Genome Anatomy Project; GEO: Gene Expression Omnibus; RT-PCR: Reverse Transcription Polymerase Chain Reaction.

## Competing interests

The authors declare that they have no competing interests.

## Authors' contributions

LJ, LX and DGL participated in the design of the study and tissues collection, optimized and carried out quantitative RT-PCR. WWZ and LJP performed data collection, in silico analysis and the statistical analysis and drafted the manuscript. CDL and DJJ also collected the tissue of gastric cancer. ZJY performed RNA extration. All authors read and approved the final manuscript.

## Pre-publication history

The pre-publication history for this paper can be accessed here:


